# Exploratory Investigation of Brain MRI Lesions According to Whole Sample and Visual Function Subtyping in Children With Cerebral Visual Impairment

**DOI:** 10.3389/fnhum.2021.765371

**Published:** 2022-01-06

**Authors:** Hanna Sakki, Naomi J. Dale, Kshitij Mankad, Jenefer Sargent, Giacomo Talenti, Richard Bowman

**Affiliations:** ^1^Developmental Neurosciences, UCL Great Ormond Street Institute of Child Health, London, United Kingdom; ^2^Neurodisability Service, Great Ormond Street Hospital NHS Foundation Trust, London, United Kingdom; ^3^Department of Radiology, Great Ormond Street Hospital NHS Foundation Trust, London, United Kingdom; ^4^Ophthalmology Department, Great Ormond Street Hospital NHS Foundation Trust, London, United Kingdom

**Keywords:** cerebral visual impairment (CVI), MRI, subtypes, visual pathway dysfunction, brain lesions, children, neuroimaging, classification

## Abstract

**Background:** There is limited research on brain lesions in children with cerebral visual impairment (CVI) of heterogeneous etiologies and according to associated subtyping and vision dysfunctions. This study was part of a larger project establishing data-driven subtypes of childhood CVI according to visual dysfunctions. Currently there is no consensus in relation to assessment, diagnosis and classification of CVI and more information about brain lesions may be of potential diagnostic value.

**Aim:** This study aimed to investigate overall patterns of brain lesions and associations with level of visual dysfunction and to compare the patterns between the classification subgroups in children with CVI.

**Methods:** School-aged children with CVI received ophthalmological and neuro-psychological/developmental assessments to establish CVI-related subtyping. Other pediatric information was collected from medical records. MRI scans were coded according to a semi-quantitative template including brain regions (right hemisphere, left hemisphere, visual pathways) and summed for total scores. Non-parametric analyses were conducted.

**Results:** 28 children had clinical brain MRI scans available [44% of total sample, Group A (lower severity of visual dysfunctions) *n* = 16, Group B (higher severity) *n* = 12]. Total brain scores ranged between 0 and 18 (Group A mdn = 7, IQR = 0.8–10.0, Group B mdn = 10, IQR = 6.5–11.8) and were widespread across regions. 71 per cent had post-geniculate visual pathway damage. The median total brain and hemisphere scores of Group B were higher than subgroup A but differences did not reach statistical significance. No statistically significant associations were found between brain scores and vision variables (acuity, contrast sensitivity).

**Conclusion:** This study found a spread of lesions across all regions on the brain scans in children with congenital CVI. The majority had damage in the postgeniculate visual pathways and visual cortex region suggesting this is an area of interest and potentially informative for diagnosis. However the subtyping classification did not show differences in number or region of lesions though the trend was higher toward Group B. This study confirms the complex diffuse and variable nature of brain lesions in children with congenital CVI, many of whom have other neurological impairments.

## Introduction

Congenital cerebral visual impairment (CVI) refers to heterogeneous visual dysfunctions of multiple pediatric etiologies that originate in the brain rather than in the ocular structures or anterior visual pathways (Sakki et al., 2018). These include disorders of acuity (discrimination of detail), visual attention, depth and motion perception, object recognition, visual perception, spatial cognition and visuo-motor integration and motor coordination skills. However knowledge of the disordered brain substrate and its relationship with the visual dysfunctions including acuity is still limited though there are exploratory advances (Merabet et al., 2017; Chang and Borchert, 2020). This study intends to consider further the relationship between brain lesions in different regions of the brain and relationship with acuity and contrast sensitivity in children with congenital CVI, using a semi-quantitative coding template for MRI brain scans (Fiori et al., 2014).

Convergent evidence suggests that congenital CVI is associated with widespread diffuse changes in the structural and functional integrity of gray and white matter pathways, including optic radiations from the optic nerves to the occipital cortex; these have been associated with reduced visual acuity and visual perceptual dysfunctions (Ortibus et al., 2012; Bauer et al., 2014; Bauer and Papadelis, 2019; Bennett et al., 2020; Pamir et al., 2021). Thalamic involvement has been associated with severe visual impairment (Cioni et al., 1996, 1997, 2000; Ricci et al., 2006; Ortibus et al., 2009; Merabet et al., 2017). Damage to occipital-parietal areas has been related to impairments in visual crowding (Drummond and Dutton, 2007; Ortibus et al., 2012). Primary visual cortex thickness has been associated with motion perception in children with periventricular leukomalacia (Bhat et al., 2021). Damage to the structure, volume and network integrity of white matter of the inferior longitudinal fasciculus has been implicated in object recognition difficulties (Ortibus et al., 2012). Performance on a task of global motion coherence was associated with integrity of the right superior longitudinal fasciculus in typically sighted children (Braddick et al., 2016) and white matter connections of the visual cortex to temporal cortex in people with CVI (Pamir et al., 2021). Using the coding template for MRI brain scans (Fiori et al., 2014), brain damage scores of global structural, hemispheric and subcortical scores were found to correlate significantly with basic vision disorder in children with cerebral palsy and periventricular leukomalacia (Tinelli et al., 2020).

CVI includes diverse individual profiles of visual disorders of differing degree of impairment which might be grouped according to classification of discrete patterns or subtypes (Fazzi et al., 2007; Boot et al., 2010; Colenbrander, 2010; Philip and Dutton, 2014). Our team has recently demonstrated that a sample of school-aged children with congenital CVI of heterogeneous etiologies had individual profiles of visual dysfunctions that could be classified into three groups; this followed data-driven cluster analysis and statistical grouped comparisons (Sakki et al., 2021). The results suggested a gradient of severity in basic and higher-level visual dysfunctions from Group A1 (least severe) to Group B (most severe) with Group A2 as intermediate, paralleled by a similar gradient in cognitive abilities and motor disorders (Sakki et al., 2021).

Using the original sample of school-aged children with congenital CVI of heterogeneous etiologies (Sakki et al., 2021), the objectives of this study were to undertake a within-group analysis of the overall patterns of brain lesions across different regions of the brain on clinical MRI brain scans and associations with level of visual dysfunction. This included comparison of these patterns between the classification subgroups. According to previous literature it was hypothesized that (1) there would be widespread and variable brain disorders within the sample according to pattern of regional sites and severity (number) of brain lesions, (2) the pattern of brain lesions would differ between the classification groups (Group A and B) with greater severity in Group B, and (3) there would be an association between visual dysfunction (acuity, contrast sensitivity) and brain disorder with greater visual dysfunction associated with greater number of brain lesions. Of exploratory interest was to consider the white matter visual pathways including the post-geniculate visual pathway and visual cortex and occipital-parietal region, with anticipation of frequent brain lesion in these pathways across the whole sample.

## Materials and Methods

### Participants

A cross-sectional observational study was conducted at the single hospital research site in a tertiary pediatric hospital including ophthalmology and developmental vision neurodisability services (research data collection conducted between 2014 and 2017). Convenience sampling recruitment was undertaken in this hospital site and in a second recruitment center in a tertiary eye hospital (with a pediatric department) and through direct parent self-referral. Details of recruitment and ascertainment and consent are available in Sakki et al. (2021) and a flow chart is available (see Supplementary Figure 1). All children were seen by the pediatric ophthalmologist (RB) prior to final decision that they met the inclusion criteria of the study. Inclusion criteria were (1) age 5–15 years, (2) a definite or provisional diagnosis (on the evidence available) of congenital CVI by RB; this was defined as the child meeting at least two of three criteria of (i) pediatric history of early brain injury/neurodevelopmental disorder, (ii) convincing symptoms of functional vision difficulties associated with CVI in daily life reported by parents, and (iii) clinical evidence of visual difficulties on examination that were considered as cerebral in origin and in the context of normal or near normal eye health. All children had an ophthalmology examination undertaken by RB to examine eye health. Exclusion criteria were (1) onset of CVI after 4 weeks after birth (according to age of condition likely to have caused the CVI such as meningitis), (2) visual difficulties caused by abnormal ocular disorder according to ophthalmologist examination (RB) and (3) parental understanding of English insufficient to complete study questionnaires.

A few children who had complex visual, motor and cognitive presentations were assessed by the neurodisability pediatrician (JS) in the multidisciplinary developmental vision neurodisability clinic. Further details of recruitment and inclusion criteria are available in Sakki et al. (2021).

#### Classification Into Subtypes

Data-driven analyses including cluster analysis of the individual profiles (according to visual acuity, contrast sensitivity, stereopsis, visual perception and visuo-motor integration scores) and statistical comparison (visual acuity, contrast sensitivity) were undertaken. This led to three subgroups (Groups A1, A2, and B), which are briefly summarized here. Group A1 (*n* = 15) had normal visual acuity and contrast sensitivity range, with selective disorder in visual perception and visuo-motor integration, Group A2 (*n* = 28) had intermediate acuity reduction (half in the mild-moderate visual impairment range and a low proportion with abnormal contrast sensitivity) and widespread disorder in visual perception and visuo-motor integration, and Group B (*n* = 20) had the most severe acuity reduction (nearly half with moderate visual impairment or worse and half with abnormal contrast sensitivity). Group B was significantly lower on visual acuity reduction than Group A and external validation showed significant co-occurring ophthalmological (e.g., strabismus) and motor impairment differences between the groups. See Sakki et al. (2021) for further details.

### Procedure

Each participant attended a single individual research assessment, where they were examined with both eyes open and best corrected vision. This consisted of basic vision and neuropsychological examination (including verbal cognition, visual perceptual, visuo-motor integration and other neuropsychological assessments, for further detail see Sakki et al., 2021) by the primary researcher (HS), supervised by the clinical neuropsychologist (ND). All assessments included standard clinical visual and psychometric methods leading to standard measurements. Separately and on a different appointment, each child underwent a clinical pediatric ophthalmology examination and orthoptic and optometric assessments (conducted by RB and an orthoptist). Medical case notes were accessed for the details and information relating to the child’s pediatric history and any related diagnoses. For this study, structural brain MRI scans available in the child’s medical reports were included with parental consent.

The routine ophthalmological examination was conducted by RB and included fundoscopy (with or without cycloplegia according to clinical need), retinoscopy, ocular motility, corneal reflexes, convergence, and visual field assessment. Optical coherence tomography was not used routinely. Fundoscopy was recorded as normal/abnormal, and presence of refractive error was coded. Nystagmus was coded as absent/present. The cover test was conducted and results for each eye recorded at near and distance as symmetrical, exotropic or esotropic. Visual fields were also assessed (see Sakki et al., 2021).

### Data Collection Instruments

#### Vision

##### Near Visual Acuity

Participants completed the recognition acuity test of Sonksen LogMAR test at 40 cm standard distance (*n* = 19; Salt et al., 2007). This pediatric optotype acuity assessment provides published developmental normative data to interpret test results up to 9 years, at which age the normative data shows stability and similar values to adult data (Sonksen et al., 2008). Test administration was terminated according to the test standard, when the child was unable to identify two sequential uncrowded optotypes or could only identify fewer than three crowded optotypes in a line. Those unable to perform this letter optotype task by matching completed the 17-card forced choice preferential looking resolution acuity grating test of Keeler Acuity Cards at 38 cm standard distance (*n* = 7, Keeler Ltd, 2014), with a reversal staircase procedure repeated five times (card 17 = 0.18 cycles per degree, approximating 2.2 logMAR, card 1 = 35.4 cycles per degree approximating –0.08 logMAR). For children who could not produce reliable responses to either test, a broad estimate of acuity level was made from near detection assessment using the Near Detection Scale (*n* = 2; Sonksen et al., 1991). Administration was terminated when the participant did not show an overt response to the presented stimulus. Scores were categorized according to the World Health Organization, as adapted by Cumberland et al. (2016; “normal”: logMAR 0.00–0.20, “near normal”: 0.21–0.30 logMAR, “socially significant VI”: 0.31–0.49 logMAR, “moderate VI”: 0.50–1.00 logMAR, “severe VI”: 1.01–1.30 logMAR, “blind”: > 1.30 logMAR).

##### Contrast Sensitivity

The Hiding Heidi Cards (Hyvärinen, 2018), a preferential looking test where the child is presented with two white 23 × 23cm cards at 1 m distance, one of which is a blank control card and the other which contains a schematic face at six contrast levels (100, 25, 10, 5, 2.5, and 1.25%) was administered with a reversal staircase procedure repeated five times. Test performance was categorized as “normal” if the participant responded correctly to the 1.25% stimulus, and “weaker” if they could not.

Other measures of higher vision including visual perception and visuo-motor integration in the main study are not included here as they were unable to be undertaken by those in Group B (with most severe visual and cognitive disability).

#### MRI Brain Scan Protocol

**FIGURE 1:**
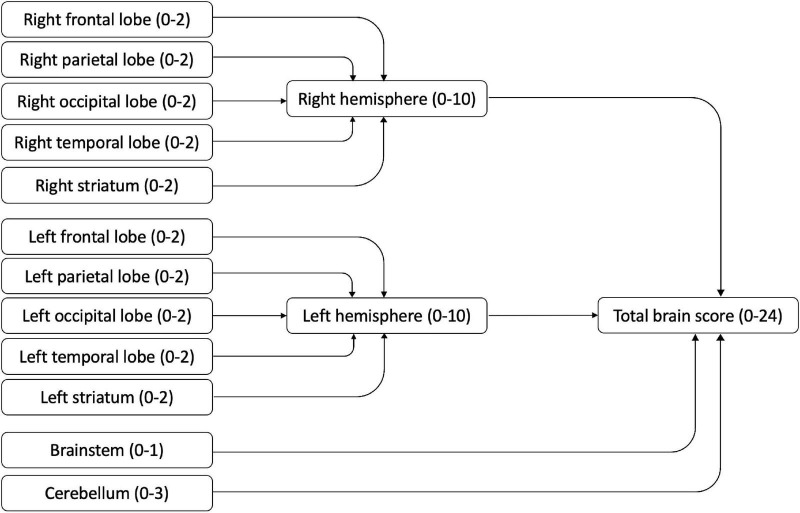
MRI scoring template (based on Fiori et al., 2014).

Children had received a clinical MRI scan in the hospital MRI scanner as part of clinical routine care. The images were performed on a 1.5T Siemens Avanto scanner, using standard imaging protocol consisting of 3D T1, Axial T2, Coronal FLAIR, DWI and ADC sequences (Saunders et al., 2007). The hospital neuroradiologist (KM) and specialist registrar (GT) scored the clinical brain MRI scans. according to a standard radiological template. Results were then coded by the researcher (HS) according to a modified version of the validated semi-quantitative template from a previous study (Fiori et al., 2014; see Figure 1). This template gives summary scores for the hemispheres (left and right, based on frontal, temporal, parietal and occipital lobes), the basal ganglia and brainstem (left and right, based on thalamus, caudate, lenticular, posterior limb of internal capsule), the corpus callosum, and the cerebellum. These summary scores can be summed for a total score. In Fiori et al. (2015) high intra- and inter-rater reliability (0.84–0.93) for the hemispheric and total global summary scores were reported. Evidence for the construct validity of this template has been reported in a sample of children with unilateral cerebral palsy, with motor and sensorimotor hand function measures correlating with contralateral lobal and hemispheric scores (Fiori et al., 2015). This coding template has since been extended to examining functional neuroimaging and white matter structures in adolescents with CVI and visual impairments related to perinatal injury (Pamir et al., 2021).

The coding template permitted investigation of brain regions separately, with higher scores indicating more damage seen on the scan. All regions were summed to obtain a total brain integrity score (range 0–24); hemisphere scores were obtained by summing the lobar and striatum scores of each hemisphere (range 0–10).

##### Cerebral Lobes

The lobes (frontal, parietal, occipital, temporal) were scored separately. For each lobe, cortical gray matter and subcortical white matter were scored (0 = no abnormality, 1 = abnormality seen) and summed for a lobar score (0–2) for each hemisphere.

##### Striatum

The thalamus and basal ganglia were coded separately (0 = no abnormality, 1 = abnormality seen) and summed for a striatum score of 0–2 in each hemisphere.

##### Brainstem

The brainstem was coded as 0 = no abnormality or 1 = abnormality seen.

##### Cerebellum

The left hemisphere, right hemisphere and vermis of the cerebellum were coded (0 = no abnormality, 1 = abnormality seen), and summed for a cerebellum score of 0–3.

In addition, the visual pathways were coded separately in this study because of the interest in vision. Three regions were considered: the pregeniculate area (optic nerves, optic tracts), postgeniculate area (optic radiations) and visual cortex. Each region was coded (0 = no abnormality, 1 = abnormality seen) and summed for a visual pathway score ranging between 0 and 3.

### Data Analysis

Data analysis was conducted using SPSS 26 (IBM Corp, 2019; RRID: SCR_002865). Non-parametric analyses were conducted due to small sample size and skewed distributions; details of tests used are given for each data table. To assess the sample representativeness of the subset with MRI scans, the subset was compared with those without scans according to general pediatric and other factors (recruitment source, weeks’ gestation at birth, age, gender, confirmation of CVI diagnosis, presence of movement disorder, verbal cognition, fundal abnormality, refractive error; Mann Whitney-*U*-tests and Chi squared tests). The brain scan scores of the classification or subtype groups (Groups A and B), were investigated and compared (Mann Whitney-*U*-tests and Fisher’s exact tests). MRI summary scores were treated as continuous variables and visual pathway scores were treated as binary/ordinal. MRI data were treated as continuous variables in all analyses. Finally, associations between brain MRI scores and vision function variables (acuity—ordinal variable, contrast sensitivity—binary variable) were investigated across the total sample (Mann Whitney-*U*-tests, Kruskal-Wallis tests). Analyses were Bonferroni corrected for four comparisons (significance level set at *p* < 0.0125).

## Results

### Descriptives

Supplementary Figure 1 gives details of participant ascertainment, numbers and reasons for exclusion and final number participating in the main study and the numbers in this study with brain scans (further details in Sakki et al., 2021). A total sample of 63 children (28 males, 35 females, median age 8 years) (Sakki et al., 2021) was recruited (58 per cent out of the 109 ascertained).

Twenty-eight children had clinical brain MRI scans available for analysis (44% of original study sample). The median age when the clinical MRI scans were undertaken was 2.61 years (*IQR* = 0.04–7.02 years). When comparing participants with and without available MRI scans, no significant differences were found in weeks’ gestation at birth, gender, whether or not the CVI diagnosis was definite or provisional (not confirmed), verbal cognition level, presence of movement disorder, presence of fundus abnormalities, refractive error (Table 1). The children with MRI scans were significantly younger and more likely to be recruited through the hospital pediatric neurodisability service; the children who were self-referred by parents into the study were less likely to have an MRI report.

**TABLE 1:** Descriptive characteristics of the study sample.

General characteristics	MRI subsample (*n* = 28)	No MRI subsample (*n* = 35)	Statistical comparison
Age at assessment (mdn, IQR)	7.65 (6.22–10.50)	10.99 (7.40–13.02)	*U* = 322, *p* = 0.02, η^2^ = 0.087
Gender (male: female)	13: 15	15: 20	χ^2^ = 0.08, *p* = 0.78, phi = -0.04
Subgroup (A1: A2: B)	3: 13: 12	12: 15: 8	χ^2^ = 2.87, *p* = 0.09, phi = 0.21
Recruitment source			
Neurodisability	1	3	χ^2^ = 21.82, *p <* 0.001, *V* = 0.59
Ophthalmology	8	9	
Self-referral	4	23	
Cognitive level, mdn (IQR)	85.5 (50.3–98)	87 (69–99)	*U* = 440, *p* = 0.49, η^2^ = 0.008
Clinical CVI diagnosis confirmed	19 (68%)	18 (51%)	χ^2^ = 1.73, *p* = 0.19, phi = 0.17
Fundal abnormality present	10 (37%, 1 missing)	8 (24%, 2 missing)	χ^2^ = 1.16, *p* = 0.40, phi = 0.14
Refractive error present	20 (71%)	18 (51%)	χ^2^ = 2.60, *p* = 0.13, phi = 0.20

**Etiological risk factor for CVI**			
Periventricular leukomalacia	9 (32%)	9 (26%)	
Intraventricular hemorrhage	6 (21%)	7 (20%)	
Likelihood of hypoxia/ischemia	6 (21%)	7 (20%)	
Neonatal infection (confirmed)	3 (11%)	5 (14%)	
Neonatal seizures	5 (18%)	1 (2%)	
Hydrocephalus	2 (7%)	3 (9%)	
Genetic (confirmed)	1 (4%)	2 (6%)	
Cerebrovascular incident	1 (4%)	2 (6%)	
Hypoglycemia	2 (7%)	1 (3%)	
Weeks’ gestation at birth, mdn (IQR)	36 (27–40)	35 (28–40)	*U = 469*, *p* = 0.96, η^2^ = 0.001

**Neurodevelopmental co-occurring condition**			
Movement disorder	17 (61%)	12 (34%)	χ^2^ = 4.37, *p* = 0.04, phi = 0.26
Current seizure disorder	5 (18%)	7 (20%)	
ASD	2 (7%)	7 (20%)	
ADHD	1 (4%)	2 (6%)	
DCD	2 (7%)	2 (6%)	
Hearing impairment	1 (4%)	1 (3%)	
Dyslexia	2 (7%)	0	

*Mdn, median; IQR, Interquartile range; U, Mann Whitney-U test; χ^2^, Chi squared test; GMFCS, Gross Motor Function Classification Scale; ASD, Autism spectrum disorder; ADHD, Attention deficit hyperactivity disorder; DCD, Developmental coordination disorder; η^2^, eta squared effect size; V, Cramer’s V effect size.*

Of the subsample with MRI scans, children were classified as Group A1 (*n* = 3), A2 (*n* = 13) and B (*n* = 12) from the original study (Sakki et al., 2021). To permit analysis in this study, Group A1 and A2 were combined to Group A (*n* = 16). There was no significant difference in frequency of whether participants were in Group A or B (χ^2^ = 1.80, *p* = 0.18, phi = 0.169).

### Region Site and Severity of Brain Lesions

Table 2 shows the MRI data for total and region scores. Participant total brain scores ranged between 0 and 18, with four children showing no apparent abnormalities (14% of MRI sample, Group A *n* = 3). Right hemisphere scores ranged between 0 and 9, left hemisphere scores ranged between 0 and 10, subcortical scores ranged between 0 and 4, and visual pathway scores ranged between 0 and 3.

**TABLE 2:** MRI brain summary scores and group comparisons.

Summary scores	Median score (IQR)		N children with no abnormality (%)		Statistical comparison
Total brain score (Score range 0–24)					
Total	8 (5–10.75)		4 (14%)		*U* = 61.5, *p* = 0.11, η^2^ = 0.092
Subgroup A	7 (0.75–10)		4 (25%)		
Subgroup B	10 (6.5–11.75)		0		

Right hemisphere score (Score range 0–10)					
Total	4 (2–5)		4 (14%)		
Subgroup A	3.5 (0.5–4.75)		4 (25%)		*U* = 66.5, *p* = 0.17, η^2^ = 0.067
Subgroup B	5 (2–5.75)		0		

Left hemisphere score (Score range 0–10)					
Total	4 (1.25–5)		6 (21%)		*U* = 70.0, *p* = 0.24, η^2^ = 0.052
Subgroup A	3.5 (0–4.75)		5 (31%)		
Subgroup B	5 (2–6.5)		1 (8%)		

**Visual pathway scores**	**0**	**1**	**2**	**3**	**Statistical comparison^a^**

Total visual pathway, n (%) (Score range 0–3)					
Total	4 (14%)	16 (57%)	7 (25%)	1 (4%)	
Subgroup A	3 (19%)	10 (63%)	2 (13%)	1 (6%)	*p* = 0.34, *V* = 0.368
Subgroup B	1 (8%)	6 (50%)	5 (42%)	0	

Pregeniculate pathway, n (%)					
Total	23 (82%)	5 (18%)			
Subgroup A	13 (81%)	3 (19%)			*p* = 1.0, phi = -0.027
Subgroup B	10 (83%)	2 (17%)			

Postgeniculate pathway, n (%)					
Total	8 (29%)	20 (71%)			*p* = 0.09, phi = 0.388
Subgroup A	7 (44%)	9 (56%)			
Subgroup B	1 (8%)	11 (92%)			

Visual cortex, n (%)					
Total	20 (71%)	8 (29%)			*p* = 1.0, phi = -0.068
Subgroup A	11 (69%)	5 (31%)			
Subgroup B	9 (75%)	3 (25%)			

*IQR, Interquartile range; U, Mann Whitney-U test, η^2^ = eta squared effect size, V = Cramer’s V effect size.*

*^a^Fisher’s exact test.*

Approximately half of children (43–54%) showed abnormalities in frontal or temporal hemispheres, or striatum (see Supplementary Table 1 for score details). Approximately three quarters of participants (71–79%) showed abnormalities in occipital or parietal areas. Cerebellar abnormality was reported in five participants (18%) and brainstem abnormality in four participants (14%).

Examining the visual pathways, five children (18%) showed an abnormality in the pregeniculate pathways, 20 (71%) showed a lesion in the postgeniculate pathway and 8 (29%) showed a visual cortex lesion. Four children (14%) showed no visual pathway abnormalities, 16 (57%) showed lesions in one area (two pregeniculate, 12 postgeniculate, two visual cortex), seven (25%) showed abnormalities in two areas (two in pregeniculate and postgeniculate pathways, five in postgeniculate pathway and visual cortex) and one child (4%) showed lesions in all three visual pathway areas.

### Comparison of Brain Lesions According to Classification Group

Table 2 reports the MRI summary scores for and group comparisons between Groups A and B. Although no statistically significant differences were found between Groups A and B in any brain regions investigated, Group A had lower median scores in all brain regions than Group B. There was a non-significant trend for more children in Group B to have postgeniculate pathway damage than in Group A (*p* = 0.09, see Table 2). Comparisons of ophthalmological impairments (nystagmus, strabismus) as a secondary analysis also did not reach statistical significance between the groups.

### Relationship Between Brain Lesions and Vision Dysfunction

The associations between region and frequency of brain lesion scores (MRI total brain, left hemisphere, right hemisphere, total visual pathway) and vision dysfunction (vision acuity or contrast sensitivity, Table 3) were low effects and did not reach statistical significance.

**TABLE 3:** Comparisons of brain summary scores with vision/eye variables.

Vision area	Categories	Total brain	Left hemisphere	Right hemisphere	Total visual pathways
Visual acuity^a^ *0 missing*	Normal (0.0–0.2) = 12 Near normal (0.21–0.3) = 2 Socially significant VI (0.31–0.49) = 2 Moderate VI (0.5–1.0) = 8 Severe VI (1.1–1.3) = 3 Blind (>1.3) = 1	ρ = 0.249 *p* = –0.201	ρ = 0.258 *p* = 0.185	ρ = 0.129 *p* = 0.512	ρ = 0.289 *p* = 0.136
Contrast sensitivity^b^ *2 missing*	Normal = 20 Weaker = 6	*U* = 48.5, *p* = 0.481, η^2^ = 0.019	*U* = 49.5, *p* = 0.518, η^2^ = 0.016	*U* = 51, *p* = 0.580, η^2^ = 0.012	*U* = 49.5, *p* = 0.481, η^2^ = 0.016
Strabismus^c^ *0 missing*	None = 4 Exotropia = 14 Esotropia = 10	*H* = 2.21, *p* = 0.331, η^2^ = 0.127	*H* = 2.64, *p* = 0.267, η^2^ = 0.107	*H* = 1.49, *p* = 0.475, η^2^ = 0.160	*H* = 2.14, *p* = 0.343, η^2^ = 0.130
Nystagmus^b^ *1 missing*	No = 15 Yes = 12	*U* = 86, *p* = 0.844, η^2^ = 0.001	*U* = 98, *p* = 0.961, η^2^ = 0.006	*U* = 76, *p* = 0.489, η^2^ = 0.017	*U* = 81.5, *p* = 0.638, η^2^ = 0.006

*^a^Spearman rank correlation.*

*^b^Mann Whitney-U test.*

*^c^Kruskal-Wallis test, η^2^ = eta squared effect size, V = Cramer’s V effect size.*

*Significance level corrected for multiple comparisons, set at p < 0.0125.*

## Discussion

This study used a standard template of brain anatomy to examine the clinical MRI scans of a sample of children with heterogeneous confirmed or provisionally diagnosed CVI. The hypothesis that there would be widespread brain disorders within the sample was confirmed. All of the sample, except four children, had identified brain disorder on their clinical MRI scans which could be classified on the semi-quantitative template of Fiori et al. (2014), highlighting that early brain damage is a clinical feature of congenital CVI. As predicted, there were variable patterns across the sample with approximately half of participants showing abnormalities in the frontal or temporal or striatum areas and approximately three quarters in the occipital or parietal areas. Cerebellar or brainstem abnormalities were present in less than a fifth of participants. The diffuse nature of the regions affected suggests that proportions of the children will have additional neurodevelopmental disorders to CVI; this was reinforced by the finding that over half of the sample have a motor disorder and the proportion below the median are in the mild-moderate intellectual disability range. This corresponds to a population-level study demonstrating that CVI-related vision problems are related to neurodevelopmental disorders, extra educational support and admission to a special care baby unit (Williams et al., 2021).

The findings are similar to previous literature describing widespread brain areas affected in children showing CVI but with a majority showing involvement of the occipital and parietal regions (Cioni et al., 1996, 1997, 2000; Drummond and Dutton, 2007; Ortibus et al., 2009; Merabet et al., 2017). The predominance of lesions in the visual pathways is as anticipated, with over four fifths of the sample showing disorder in some aspect of the visual pathway. Nearly three quarters showed damage in the postgeniculate pathways and over a quarter showed lesions in the visual cortex. Very few children showed lesions in the pregeniculate pathways, confirming that the visual dysfunctions were not attributed to disorders of the anterior visual pathways (Fazzi et al., 2007; Good, 2007; Colenbrander, 2010; Philip and Dutton, 2014). However we found some degree of pregeniculate involvement; this is not uncommon in CVI (Fazzi et al., 2007; Good, 2007; Philip and Dutton, 2014) with ocular features of nystagmus and strabismus quite prevalent in this study (Sakki et al., 2021). Very few children showed absence of visual pathway abnormalities and therefore consideration of these pathways, particularly the postgeniculate pathways, is likely to be of clinical radiological importance that may also be informative for diagnosis.

The second hypothesis that the pattern of brain lesions would differ between the classification groups was inconclusive. As postulated, Group B (with more severe acuity reduction) had a higher number of lesion sites in the total score and each brain region than Group A, but these differences did not reach statistical significance. The median total brain and hemisphere scores of Group B were higher than Group A. Over ninety per cent of Group B showed damage in the postgeniculate visual pathways, in contrast to only half of Group A, but this difference failed to reach statistical significance. All of Group B had some degree of structural abnormality and spread across all brain regions, whereas a quarter of children in Group A had no brain abnormalities visible on MRI. Together these findings point suggestively toward greater severity of visual disorder and higher prevalence of other neurodevelopmental conditions in Group B (Sakki et al., 2021) but more research with a larger sample is needed in the future. As in Ortibus et al. (2009) and Goodale (2013), there was a small proportion of children found in Group A who had visual perceptual dysfunction on test scores but had normal structural MRI scans.

Although our small sample size precluded comparing brain lesion patterns within Group A (Groups A1 vs. A2), our previous data driven cluster analysis showed that in the; “least severe” group (A1) the visual perceptual disorders tended to be more visuo-motor in nature and potentially indicating “dorsal stream impairment” (Sakki et al., 2021). This would be expected to be associated with occipito-parietal impairments; as three quarters of our sample showed lesions in these regions it is postulated that these brain regions are of significance and diagnostic relevance (Ffytche et al., 2010) but we were unable not compare Group A1 and A2 in this study. More recent interest in adult visual perceptual disorders is exploring how each higher visual function relates to a specific network of cortical locations and white-matter connections which may be *topological* (localized within a cortical area) or *hodological* (connections between areas and where function in one region is altered by changes in another spatially remote region, Ffytche et al., 2010).

In relation to the final hypothesis of an association of brain lesions with visual dysfunction, this was not supported by our evidence. No clear associations were found between regions or numbers of brain lesions and degree of visual acuity and contrast sensitivity. This contrasts with the findings of Tinelli et al. (2020) who reported strong correlations between global brain lesion severity on MRI scans and visual function impairment scores in children with cerebral palsy and periventricular leukomalacia using a similar MRI coding template to the template used in this study. One noteworthy difference is that Tinelli et al. used a single encompassing category of “visual dysfunction” including fixation, saccades, nystagmus, acuity, visual field, stereopsis, color perception, whereas our current study investigated only acuity and contrast sensitivity separately. Our secondary group comparisons of ophthalmological features (nystagmus, strabismus) showed no significant differences. Our study only focused on acuity and contrast sensitivity but it is well established that there are different anatomical substrates for specific aspects of visual dysfunction, such as object recognition impairment with normal acuity but probably a posterior cortical lesion or visual acuity reduction and only a subcortical lesion (Cioni et al., 2000; Ricci et al., 2006; Ortibus et al., 2009).

We were unable to investigate visual perceptual dysfunction across the sample as only the children in Group A had sufficient acuity to undertake these test activities (Sakki et al., 2021). Differences in our findings with Tinelli et al. may result from their focus on a single clinical population and brain lesion site (periventricular leukomalacia) leading to a strong correlation with vision function whereas our study had multiple etiologies and brain lesion sites and no significant correlation with vision.

The evidence of our study suggests diverse widespread and diffuse brain involvement in children who have CVI of heterogeneous etiologies, but many in this sample have other neurodevelopmental impairments too and it is not known whether some of the brain lesion abnormalities are causal or incidental to the visual dysfunctions. Of theoretical interest are the high proportion with postgeniculate visual pathway and/or visual cortex damage and the high proportion with lesions in the occipital-parietal regions. The brain scans were undertaken when the children were on average 2 years old but the visual function data was assessed when the children were school age (broad range of 6–10 years). Early neuroplastic and ongoing neurodevelopmental growth and compensatory processes (such as language development and verbal mediation) may enable some children to have improving acuity or better functional use of available vision (Fazzi et al., 2007; Good, 2007; Guzzetta et al., 2010; Merabet et al., 2017; Pamir et al., 2021). This might explain why Group A and Group B did not differ significantly in brain lesions but other protective factors may be at play in the improved visual acuity levels of Group A.

Clinical structural MRI brain scans may not be sensitive enough to identify differences in white matter connectivity affecting selective visual attention and motion vision which has been shown at experimental research level. Our findings are similar to previous research suggesting that the ability to predict functional outcomes of CVI from the clinical neuroimaging are not at a reliable level (Jacobson et al., 2006; Boot et al., 2010; Chang and Borchert, 2020). However, as neuroimaging techniques develop further, the role of brain anatomical classification may become more relevant (Bauer et al., 2014; Merabet et al., 2017; Bennett et al., 2020; Pamir et al., 2021).

This study analysis had limitations which affects generalizability of findings. Firstly, although the study investigates children with CVI due to different etiologies, the total number of subjects is very small considering the different rare etiologies. A larger heterogeneous sample is needed in the future with adequate sampling of different heterogeneous etiologies to ensure that one group, such as cerebral palsy, is not skewing the data. Secondly, of the sample of 28 children, only 16 were in Group A and of these four had a normal MRI scan and some subjects enrolled were of provisional diagnosis by the ophthalmologist (and not clinically confirmed by the multidisciplinary team). The variance in Group A may have been skewed by enrollment decisions and the smaller sample size (further divided into two small subgroups) may be underpowered to consider anatomical differences between the subgroups. It is notable that *post hoc* power calculation indicated that a sample of approximately *n* = 90 would lead to statistically significant group differences in the postgeniculate visual pathways. The merging of Group A1 and A2, which were identified in our original study through cluster analysis, may have obscured important variance between the groups. Thirdly, there may have been sampling biases as the MRI scans were undertaken for medical need and children with the MRI scans were skewed to the younger age range and recruited through hospital routes. Although it did not reach statistical significance, nearly two thirds of the MRI sample had movement disorder compared to less than a third of the non-MRI sample, suggesting that this clinical group were more likely to have early brain scans. However, the main etiological factors of periventricular leukomalacia, intraventricular hemorrhage and likelihood of hypoxia/ischemia were of similar proportions as in the original total sample. Fourthly, the high level of pediatric co-occurring conditions and diverse etiologies of CVI with known broader impacts on brain structure (e.g., cerebral palsy, hydrocephalus, cerebrovascular events) may have complicated the analysis and interpretation of the brain lesion patterns. Finally, the study was limited in considering only two and not necessarily the most important aspects of visual dysfunction in CVI—visual acuity and contrast sensitivity; this restriction reflected the only visual measures that could be undertaken across both Group A and B for comparison purposes. The main study included major other areas of visual function including stereopsis, field, visual perception, visuo-motor integration and visual attention, which could not be included in this paper.

This study has demonstrated that there is widespread brain disorder in a heterogeneous sample of school-aged children with definite or provisional diagnosed CVI and that use of a quantitative MRI template (with additional focus on the visual pathways and visual cortex) is a useful means of quantifying and grouping brain lesions in this population. Further analyses according to the classification or subtyping of the sample using the methods of Sakki et al. (2021) would be usefully considered with a larger powered sample and a prospective brain imaging protocol. The multidisciplinary integrated assessment involving ophthalmological, neurodisability pediatrics and neuropsychological assessment of basic and higher visual functions in the context of a neurodevelopmental framework was able to assess each child’s functional profile, which formed the basis for the data-driven analyses and the group classifications. This method could be suitably used in the clinic for identifying and diagnosing CVI and establishing the child’s individual profile and clinical and habilitation needs. Further research with larger samples of school-aged children with CVI of heterogeneous etiologies or single clinical populations such as cerebral palsy will be valuable to build on these findings.

## Data Availability Statement

The raw data supporting the conclusions of this article will be made available by the authors, without undue reservation.

## Ethics Statement

The studies involving human participants were reviewed and approved by the Fulham National Health Service Research Ethics Committee. Written informed consent to participate in this study was provided by the participants’ legal guardian/next of kin.

## Author Contributions

HS, ND, RB, and JS contributed to conception and design of the study. HS organized the database, performed all other data analyses, and wrote the first draft of the manuscript. HS and JS modified the MRI coding template. KM and GT analyzed the MRI data. ND contributed to the second and final drafts. All authors contributed to manuscript revision, read, and approved the submitted version.

## Conflict of Interest

The authors declare that the research was conducted in the absence of any commercial or financial relationships that could be construed as a potential conflict of interest.

## Publisher’s Note

All claims expressed in this article are solely those of the authors and do not necessarily represent those of their affiliated organizations, or those of the publisher, the editors and the reviewers. Any product that may be evaluated in this article, or claim that may be made by its manufacturer, is not guaranteed or endorsed by the publisher.
