# Supplementary material for: Exploratory Investigation of Brain MRI Lesions According to Whole Sample and Visual Function Subtyping in Children With Cerebral Visual Impairment
Source: Front Hum Neurosci. 2022 Jan 6;15:765371. doi: 10.3389/fnhum.2021.765371 (PMC8770951; doi:10.3389/fnhum.2021.765371)
Supplement: Supplementary file 1 [file Data_Sheet_1.pdf]

Supplementary Table 1: Full MRI brain scores in total sample, subgroup A and subgroup B

**Right lobar scores**	**0**	**1**	**2**	
Frontal, n (%)				
Total	15 (54%)	10 (36%)	3 (11%)	
Subgroup A	9 (56%)	6 (38%)	1 (6%)	
Subgroup B	6 (50%)	4 (33%)	2 (17%)	
Parietal, n (%)				
Total	7 (25%)	17 (61%)	4 (14%)	
Subgroup A	5 (31%)	10 (63%)	1 (6%)	
Subgroup B	2 (17%)	7 (58%)	3 (25%)	
Temporal, n (%)				
Total	16 (57%)	9 (32%)	3 (11%)	
Subgroup A	10 (63%)	5 (31%)	1 (6%)	
Subgroup B	6 (50%)	4 (33%)	2 (17%)	
Occipital, n (%)				
Total	6 (21%)	13 (46%)	9 (32%)	
Subgroup A	5 (31%)	6 (38%)	5 (31%)	
Subgroup B	1 (8%)	7 (58%)	4 (33%)	
Striatum				
Total	14 (50%)	11 (39%)	3 (11%)	
Subgroup A	10 (63%)	4 (25%)	2 (13%)	
Subgroup B	4 (33%)	7 (58%)	1 (8%)	
**Left lobar scores**	**0**	**1**	**2**	
Frontal, n (%)				
Total	13 (46%)	11 (39%)	3 (14%)	
Subgroup A	8 (50%)	7 (44%)	1 (6%)	
Subgroup B	5 (42%)	4 (33%)	3 (25%)	
Parietal, n (%)				
Total	8 (29%)	15 (54%)	5 (18%)	
Subgroup A	6 (38%)	8 (50%)	2 (13%)	
Subgroup B	2 (17%)	7 (58%)	3 (25%)	
Temporal, n (%)				
Total	14 (50%)	10 (36%)	4 (14%)	
Subgroup A	8 (50%)	6 (38%)	2 (12.5%)	
Subgroup B	6 (50%)	4 (33%)	2 (17%)	
Occipital, n (%)				
Total	7 (25%)	15 (54%)	6 (21%)	
Subgroup A	5 (31%)	8 (50%)	3 (19%)	
Subgroup B	2 (17%)	7 (58%)	3 (25%)	
Striatum				
Total	13 (46%)	10 (36%)	5 (18%)	
Subgroup A	10 (62%)	3 (19%)	3 (19%)	
Subgroup B	3 (25%)	7 (58%)	2 (17%)	
**Other brain areas**	**0**	**1**	**2**	**3**
Cerebellum, n (%) (score range 0-3)				
Total	23 (82%)	3 (11%)	0	2 (7%)
Subgroup A	14 (88%)	2 (13%)	0	0
Subgroup B	9 (75%)	1 (8%)	0	2 (17%)
Brainstem (score range 0-1)				
Total	24 (86%)	4 (14%)		
Subgroup A	15 (94%)	1 (6%)		
Subgroup B	9 (75%)	3 (25%)		

Supplementary Figure 1: Flow chart of participant ascertainment

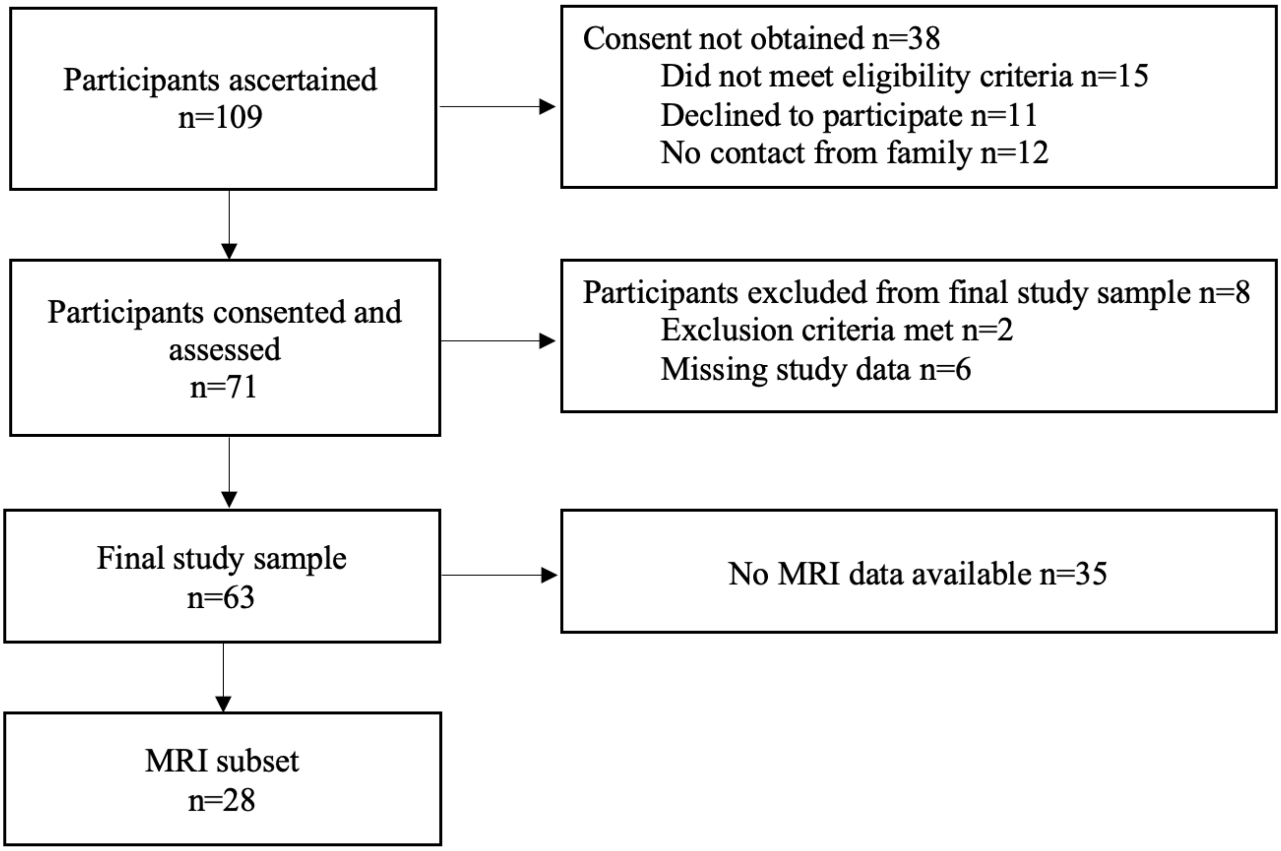

Note. Further details of the participant ascertainment process can be found in Chapter 4 of Sakki, 2018, unpublished thesis (available from https://discovery.ucl.ac.uk/id/eprint/10063803/).
